# In Silico Genomic Analysis of Antibiotic Resistance Genes Carried by Mobile Genetic Elements in *Pseudomonas aeruginosa*

**DOI:** 10.3390/ijms27135938

**Published:** 2026-07-01

**Authors:** Yang Liu, Yiye Han

**Affiliations:** 1 Department of Microbiology, School of Medicine, Zhejiang University, Zhejiang 310058, China; 2 School of Life Sciences, Westlake University, Hangzhou 310024, China; hanyiye@westlake.edu.cn

**Keywords:** *Pseudomonas aeruginosa*, antibiotic resistance genes, mobile genetic elements, plasmids, integrative and conjugative elements, horizontal gene transfer

## Abstract

*Pseudomonas aeruginosa* is a notable opportunistic pathogen in the ESKAPE group due to its multidrug resistance (MDR) and its ability to cause severe healthcare-associated infections. Horizontal gene transfer (HGT) facilitates the dissemination of antibiotic resistance genes (ARGs) through mobile genetic elements (MGEs). A comprehensive genomic analysis of ARGs associated with these elements is essential to understand multidrug resistance in *P. aeruginosa*. Here, we analyzed 10,412 publicly available *P. aeruginosa* genome assemblies defined by the Genome Taxonomy Database (GTDB, release 226) species cluster, which provides standardized prokaryotic genome taxonomy. We identified plasmids, prophages, integrative and conjugative elements (ICEs), and integrative and mobilizable elements (IMEs) carrying ARGs. A group of highly prevalent ARG families was identified in *P. aeruginosa*, comprising *mexD*, *fosA*, *catB7*, *blaPAO*, and *aph(3′)-IIb*, each of which was detected in over 96% of the genome assemblies. In contrast, 313 ARG families were found in fewer than 20% of the genomes. Many ARGs were located on plasmids, with certain pairs co-occurring frequently, such as *aph(3″)-Ib* and *aph(6)-Id*, CmlA9 and *aadA6*, or *aac(6′)-Ib3* and *aph(3′)-XV*, which were associated with specific plasmids. Some of these plasmids closely resembled plasmids from *E. coli* and *K. pneumoniae*. Moreover, other MGEs displayed distinct ARG cargo enrichment: *mexD* on IMEs, *aph(3′)-IIb* on prophages, and *sul1*, *fosA*, and *catB7* on ICEs. Our study provides a high-resolution map of the *P. aeruginosa* MGE resistome and highlights the potential roles of MGEs in disseminating different resistance genes. Our results emphasize the significance of ICE- and plasmid-associated ARG dissemination, particularly *sul1*, which may be linked to class 1 integrons. They also suggest that interspecies plasmid exchange may contribute to the evolution of MDR in *P. aeruginosa*.

## 1. Introduction

*Pseudomonas aeruginosa* is a major opportunistic pathogen and a prominent member of the ESKAPE group, which also includes *Enterococcus faecium*, *Staphylococcus aureus*, *Klebsiella pneumoniae*, *Acinetobacter baumannii*, and *Enterobacter* species [1]. These pathogens are recognized for their ability to evade the effects of many conventional antibiotics and represent some of the most critical threats in healthcare-associated infections [2]. *P. aeruginosa* is particularly associated with severe respiratory infections, including ventilator-associated pneumonia and chronic lung colonization in patients with cystic fibrosis, and continues to contribute substantially to global morbidity and mortality from respiratory diseases [3].

Clinical management of *P. aeruginosa* infections relies on a limited set of antibiotics, including carbapenems, cephalosporins, aminoglycosides, and fluoroquinolones [4,5]. However, the increasing prevalence of antibiotic resistance has greatly constrained these therapeutic options [6]. Resistance to these antibiotics is mediated by a variety of well-characterized mechanisms. β-Lactam resistance is commonly driven by chromosomal AmpC β-lactamase (*blaPAO*) and acquired β-lactamases, such as extended-spectrum β-lactamases and carbapenemases, which reduce the efficacy of cephalosporins and carbapenems [7]. Aminoglycoside resistance is often caused by aminoglycoside-modifying enzymes, including acetyltransferases, phosphotransferases, nucleotidyltransferases, and 16S rRNA methyltransferases, which impair drug binding to the ribosome [7]. Additionally, multidrug efflux systems, particularly Mex family pumps (e.g., MexAB-OprM), contribute to resistance against multiple antibiotic classes [8]. Overexpression of the MexCD-OprJ efflux system has also been associated with decreased susceptibility to a broad range of antimicrobial agents [8,9]. The emergence and dissemination of antibiotic resistance genes (ARGs) are largely driven by horizontal gene transfer (HGT), in which mobile genetic elements (MGEs) play a central mediating role [10,11]. MGEs encompass plasmids, integrative and conjugative elements (ICEs), integrative and mobilizable elements (IMEs), and prophages, all of which contribute to the acquisition and spread of resistance across bacterial populations [12,13].

Historically, plasmids have been the primary focus of research due to their high mobility and frequent carriage of multiple resistance genes [14,15,16,17,18,19]. Recent studies have expanded this view by highlighting the role of ICEs and IMEs as additional reservoirs and vehicles of resistance genes in *P. aeruginosa* [20]. Nevertheless, current understanding remains limited in several key aspects. Many studies are based on small datasets of experimentally isolated strains or combine multiple species, which can obscure species-specific trends [11,21]. Comprehensive analyses based on large publicly available genome datasets remain rare, leaving much of the global diversity of resistance genes uncharacterized [22,23]. Furthermore, most previous studies do not distinguish between ARGs located on MGEs and those in the chromosomal background, which is essential for understanding the potential for horizontal dissemination [11,24].

To address these gaps, we performed a systematic, large-scale analysis of ARGs carried by diverse MGEs in *P. aeruginosa*, including plasmids, prophages, ICEs, and IMEs. Our study included 10,412 publicly available genome assemblies classified as *P. aeruginosa* according to the Genome Taxonomy Database (GTDB) release 226, including both complete and draft assemblies.

## 2. Result

### 2.1. Distribution of ARGs in P. aeruginosa

**Figure 1:**
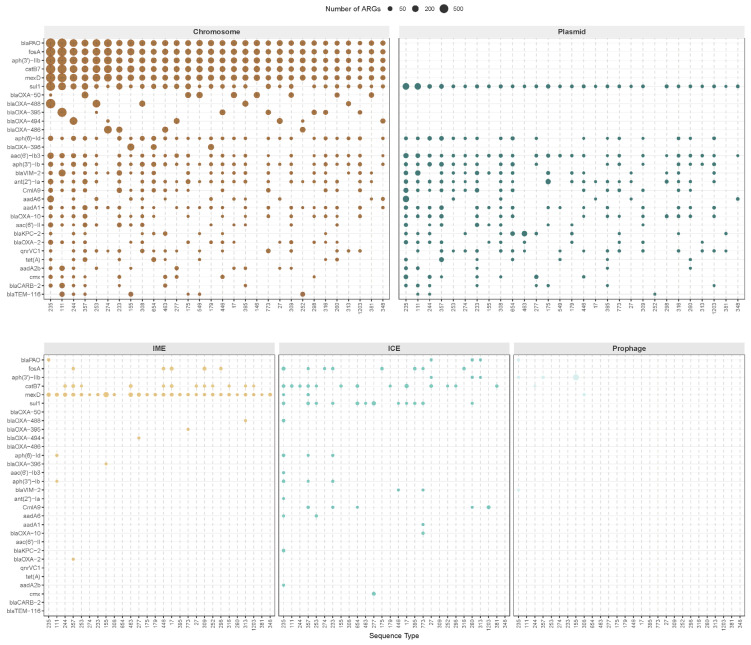
Distribution of ARG abundance across sequence types (STs) and genomic locations in *P. aeruginosa*. STs are shown on the *x*-axis and ordered by the number of genomes assigned to each ST, from highest to lowest abundance. Genomes without assigned STs were excluded from this analysis. Only the 30 most abundant STs and 30 most abundant ARG families are shown.

We systematically analyzed 10,412 publicly available *P. aeruginosa* genome assemblies (Supplementary Table S1) classified according to GTDB release 226; a total of 672,074 genes were predicted as ARGs by RGI, and 83,706 genes were predicted as ARGs by ResFinder. Of the ARGs predicted by both RGI and ResFinder, we identified 81,416 genes from 10,401 genomes across 320 ARG families for downstream analyses. Here, each gene was counted in every genome it appeared in, so the same ARG in different genomes was counted multiple times. Among these genes, *blaPAO* was the most abundant ARG family, followed by *fosA*, *aph(3′)-IIb*, *catB7* and *mexD* (Figure 1, Supplementary Figure S1A and Table S2).

We then assessed the prevalence of these ARGs across *P. aeruginosa* genomes. *mexD*, *fosA*, *catB7*, *blaPAO*, and *aph(3′)-IIb* were present in over 96% of the genomes (Supplementary Figure S1B and Table S3), indicating that these ARG families are highly prevalent and widely distributed in *P. aeruginosa*. In contrast, members of the *sul* and *blaOXA-50* families were found in 27.82% and 23.74% of genomes, respectively (Figure S1B). Moreover, 313 ARG families were detected in fewer than 20% of the genomes (Supplementary Table S3), including *blaOXA-494*, *blaOXA-488*, *blaOXA-486*, *blaOXA-396*, *blaOXA-395*, *aph(6)-Id*, *aph(3″)-Ib*, suggesting that many less prevalent ARGs may be variably distributed and could include horizontally transferable ARG families.

Using multilocus sequence typing (MLST), 9743 of the 10,412 genomes (93.6%) were assigned to known sequence types (STs). Then, we examined the distribution of ARG families across STs and genomic contexts. The five most prevalent ARG families *blaPAO*, *fosA*, *aph(3′)-IIb*, *catB7*, and *mexD* were mainly located on chromosomes across most STs (Figure 1 and Supplementary Table S4). However, these ARGs were also found in some MGEs, such as ICEs, IMEs, and prophages. For instance, *aph(3′)-IIb* was found in prophage regions of ST155 genomes (Figure 1). The *sul1* gene was primarily detected on plasmids across multiple STs, though several STs harbored *sul1* on ICEs as well (Figure 1). In contrast, *mexD* was detected on IMEs in nearly all STs, in addition to its predominant chromosomal localization (Figure 1). Less prevalent ARG families were mainly found on chromosomes and plasmids, with only rare occurrences in other genomic regions.

**Figure 2:**
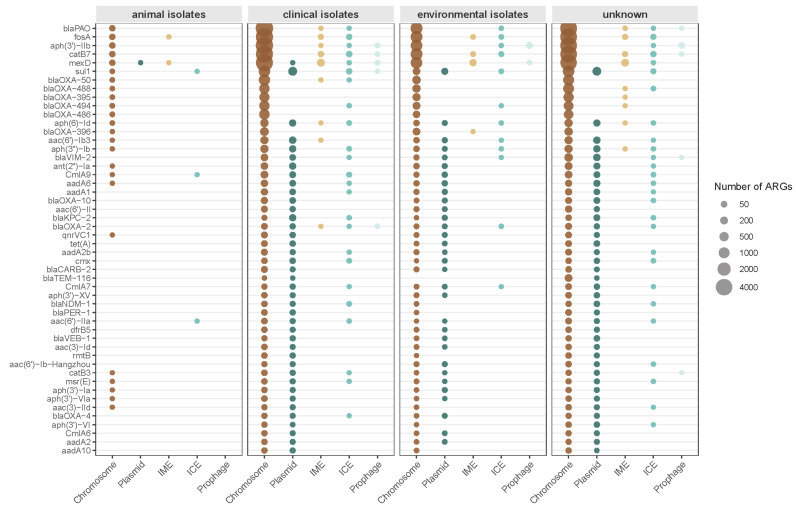
Distribution of ARG abundance across clinical, environmental, and animal isolates and genomic locations. Only the 50 most abundant ARG families are shown.

Based on NCBI isolation source metadata, 4658 genomes were classified as clinical isolates, 1406 as environmental isolates, and 42 as animal isolates, whereas 4295 genomes could not be assigned to a specific category. Overall, clinical isolates showed a similar pattern to environmental isolates (Figure 2). In contrast, animal isolates harbored a more limited diversity of ARGs, which may be associated with their smaller sample size. In addition, *mexD* homologs were detected in diverse genomic contexts, including plasmids, ICEs, IMEs, and prophage regions in clinical isolates (Figure 2 and Supplementary Table S5), although *mexD* is typically considered a chromosomally encoded efflux pump gene in *P. aeruginosa*.

### 2.2. Plasmid-Borne ARGs in P. aeruginosa

**Figure 3:**
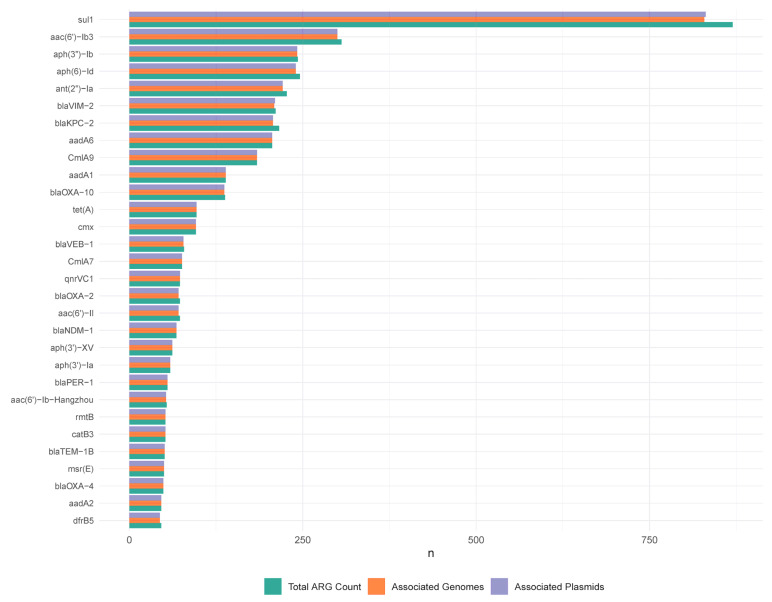
Distribution and abundance of plasmid-borne ARGs in *P. aeruginosa*. The green bars indicate the total number of ARGs in each ARG family located on plasmids. The orange bars show the number of genomes carrying at least one corresponding ARG. The purple bars represent the number of plasmid units carrying at least one corresponding ARG.

Across the *P. aeruginosa* genomes, we identified 5810 ARGs belonging to 224 ARG families located on plasmids (Supplementary Table S6). We ranked plasmid-borne ARG families by total gene count, the number of genomes carrying them, and the number of plasmids on which they occurred. The ARG families that are nearly ubiquitous in *P. aeruginosa* genomes, such as *blaPAO*, *aph(3′)-IIb*, *catB7*, and *fosA*, were not detected on plasmids in our dataset (Figure 2 and Figure 3), supporting their predominant chromosomal localization. In contrast, the frequently detected plasmid-borne ARG families were associated with several clinically relevant antibiotic classes. Plasmid-borne β-lactamase genes included carbapenemase genes, such as *blaVIM-2*, *blaNDM-1*, and *blaKPC-2*, which can confer resistance to carbapenems and other β-lactam antibiotics [25]. Aminoglycoside resistance genes included *ant*, *aac*, *aph*, *aad*, including *ant(2″)-Ia* (resistant to gentamicin and tobramycin) and *aac(6′)-Ib* (resistant to amikacin/tobramycin) [26]. *sul1* mediates sulfonamide resistance and is a signature gene of class 1 integrons, which can be embedded in chromosomes or horizontally transferred via plasmids [11,27].

The *sul1* gene family was the most abundant and widely distributed on plasmids (Figure 3), followed by *aac(6′)-Ib3*, *aph(3″)-Ib*, *aph(6)-Id*, *ant(2″)-Ia*, *blaVIM-2*, *aadA6*, CmlA9, *aadA1*, and *blaOXA-10*, each occurring in at least 137 genomes, corresponding to ≥1.32% of *P. aeruginosa* genomes. Specifically, plasmid-borne *sul1* was present in 8% of total genomes and 12.13% of genomes that had plasmids (Figure 3 and Supplementary Table S6). Given that *sul1* was detected in 27.82% of genomes overall, these results suggest that a substantial subset of *sul1*-positive genomes carried plasmid-associated *sul1*.

**Figure 4:**
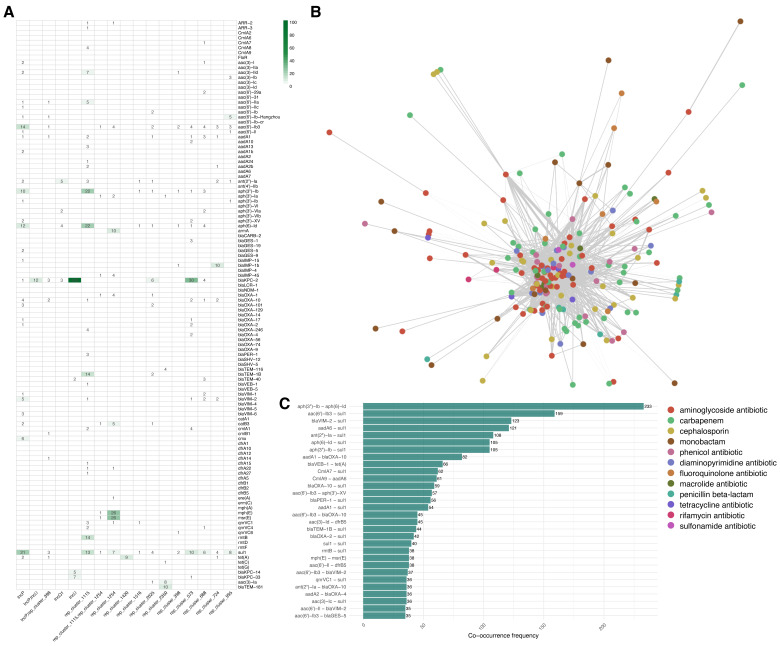
Co-occurrence of plasmid-borne ARGs and plasmid backbones in *P. aeruginosa*. (**A**) Number of ARGs from each ARG family located on plasmid backbones, represented by replicon types. (**B**) Co-occurrence network of plasmid-borne ARGs, where nodes represent ARG families and edges represent co-location on the same plasmid; edge weight corresponds to the number of plasmids where each ARG pair is co-located. (**C**) Distribution of edge weights from panel B, showing the top 30 most frequent co-occurring ARG pairs.

We annotated plasmids according to their known backbone replicon. Of the 5810 ARGs detected on plasmids, 784 were located on plasmids that had replicon annotation (Figure 4A and Supplementary Table S7). The most frequent gene was *blaKPC-2*, with 103 copies located on IncU plasmids, suggesting that IncU plasmids are important carriers of *blaKPC-2*-associated β-lactam resistance in *P. aeruginosa*. Additionally, 30 copies of *blaKPC-2* were found on rep_cluster_573 plasmids (clustered by MOB-suite), and 12 copies on plasmids with IncP-IncU replicons (Figure 4A). Among IncP plasmids, there were 21 *sul1*, 14 *aac(6′)-Ib3*, 12 *aph(6)-Id*, and 10 *aph(3″)-Ib* genes, suggesting that IncP plasmids are recurrent carriers of aminoglycoside resistance genes. Finally, many plasmid types clustered by MOB-suite carried *sul1* (Figure 4A), showing that the *sul1* gene family was not restricted to a single plasmid backbone.

### 2.3. Plasmid-Borne ARG Co-Occurrence Network in P. aeruginosa

Although most plasmids lack backbone replicon annotation, patterns of multidrug resistance dissemination can be explored through the co-occurrence of ARGs on the same plasmid. To this end, we constructed a co-occurrence network in which nodes represent ARGs, and edges represent their presence on individual plasmids with edge weights indicating the number of ARG pairs co-located on a plasmid (Supplementary Table S8). The gene families were mainly distributed among aminoglycoside, carbapenem, and cephalosporin associated genes, accounting for 25.89%, 20.54%, and 13.36% of all gene families, respectively.

Most aminoglycoside resistance genes were connected near the center of the network, indicating that they frequently co-occur with other ARGs on plasmids in *P. aeruginosa*. Specifically, *aph(3″)-Ib* co-located with *aph(6)-Id* on the same plasmid 233 times, and *aac(6′)-Ib3* co-located with *aph(3′)-XV* 57 times. In contrast, most carbapenem resistance genes were located at the periphery of the network, suggesting that they co-occur less frequently with two or more other ARG families on the same plasmid. Additionally, *aadA1* co-occurred frequently with *blaOXA-10* (82 times) and *tet(A)* with *blaVEB-1* (66 times). CmlA9 and *aadA6* also co-occurred 61 times. Notably, *sul1* was located at the center of the network with 165 connected edges (Figure 4B), likely due to it frequently co-occurring with a wide range of other resistance genes on the same plasmids (Figure 4B,C). Plasmids carrying *sul1* tend to harbor multiple ARGs, which is consistent with previous findings [11,27,28].

**Figure 5:**
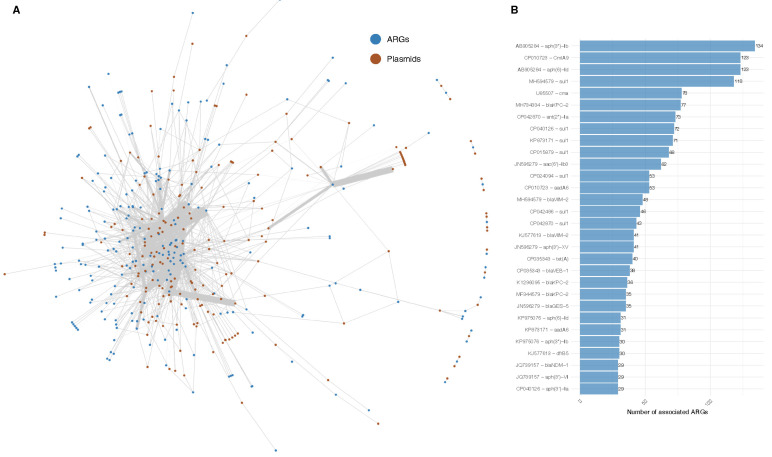
Bipartite network of plasmid-borne ARGs and plasmids in *P. aeruginosa*. (**A**) Association between plasmid types and ARG families. Nodes representing ARGs correspond to ARG families, and nodes representing plasmids correspond to representative plasmids in the MOB-suite database that are the nearest neighbors of reconstructed plasmids in our dataset based on Mash distance. Edge thickness indicates the number of plasmid–ARG pairs. (**B**) Distribution of edge weights from panel A, showing the top 30 most frequent ARG–plasmid associations.

To further investigate whether certain ARGs were associated with specific plasmids, we constructed bipartite networks linking plasmid types and ARG families (Figure 5A and Supplementary Table S9). Since most plasmids lack replicon or backbone annotation, we selected the nearest neighbor in the database based on Mash distance as a proxy. We found that previously co-located *aph(3″)-Ib* and *aph(6)-Id* were associated with plasmids that resembled pER24y-8ksm (AB905284) (Figure 5A,B). The co-located CmlA9 and *aadA6* were associated with plasmids that resembled pP18_h (CP010723) (Figure 5A,B). The co-located *aac(6′)-Ib3* and *aph(3′)-XV* pair was associated with plasmids resembling pINCan01 (JN596279) (Figure 5A,B). These ARGs frequently co-occurred on similar plasmids, which may reflect shared plasmids contributing to their co-dissemination. Additionally, *sul1* was present together with many ARG families on multiple plasmid types identified in the MOB-suite database (Figure 5B).

### 2.4. Prophage-, ICE-, and IME-Borne ARGs in P. aeruginosa

In addition to plasmids, studies have shown that ICEs and IMEs can also mediate HGT via conjugation, either autonomously or with the assistance of other conjugative elements [29,30,31]. Although these elements are integrated into the chromosome, the ARGs they carry do not represent chromosomally stabilized, vertically inherited resistance genes; rather, they are prone to frequent horizontal transfer along with the MGEs [11,29,30].

**Figure 6:**
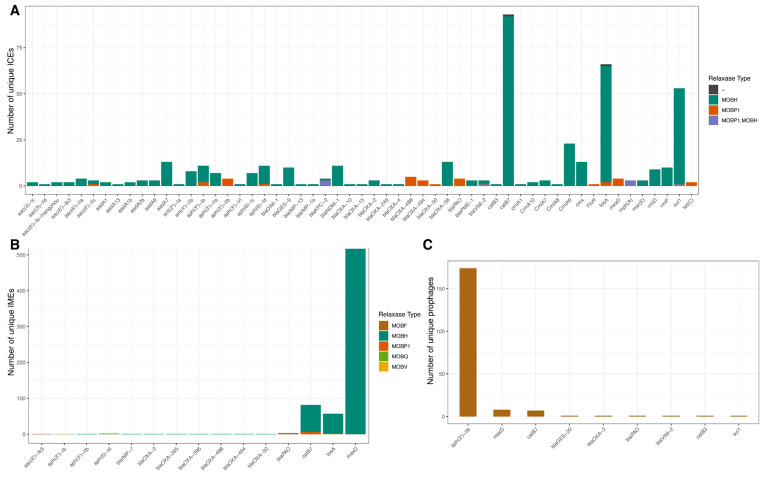
Distribution of ARG families on ICEs, IMEs, and prophages in *P. aeruginosa*. (**A**) Number of ICEs harboring each ARG family. (**B**) Number of IMEs harboring each ARG family. (**C**) Number of prophages harboring each ARG family.

To this end, we quantified ARGs located on ICEs and IMEs and found that the most frequently detected ARG families on ICEs were *catB7* (detected in 93 ICEs), followed by *fosA* (detected in 66 ICEs) and *sul1* (detected in 53 ICEs) (Figure 6A), with *fosA* being the most common gene of fosfomycin resistance. Additionally, five other ARG families (CmlA9, *aadA7*, *blaOXA-56*, *cmx*, and *blaNDM-1*) were present in over 10 ICEs (Figure 6A and Supplementary Table S10). Most ICE-associated ARGs were located on elements assigned to the MOBH relaxase type, and a similar pattern was observed for IME-associated ARGs. Three ARG families on IMEs were present in more than 50 instances: *mexD*, *catB7*, and *fosA*, with *mexD* being the most prevalent, detected in 517 IMEs (Figure 6B).

Prophages have also been recognized as mediators of ARG dissemination [32,33]. In *P. aeruginosa* prophages, we identified nine ARG families, typically carrying only a single ARG per prophage. Eight of these ARGs were found in fewer than 10 prophages (Figure 6C), corresponding to less than 0.1% of the analyzed genomes. *aph(3′)-IIb* was present in 174 prophages (Figure 6C), accounting for approximately 1.67% of genomes. This gene confers resistance to aminoglycosides such as gentamicin and tobramycin. No ARGs were detected on phage satellites in our dataset.

In summary, prophages, ICEs, and IMEs all contribute to the carriage and potential dissemination of ARGs in *P. aeruginosa*, with element-specific enrichment: *mexD* on IMEs, *aph(3′)-IIb* on prophages, and *sul1*, *fosA*, and *catB7* on ICEs.

## 3. Discussion

In this study, we identified ARGs located on both the chromosomal background and MGEs of *P. aeruginosa*, with a particular focus on ARGs carried by MGEs, because MGEs can serve as vectors for HGT. Using comparative genomics and network construction approaches, we observed that MGE-associated ARGs showed element-specific distribution patterns, meaning that particular types of ARGs tend to be associated with specific types of MGEs. For example, *mexD* is often found on IMEs, *aph(3′)-IIb* on prophages, and *sul1*, *fosA*, and *catB7* on ICEs. Notably, ARG pairs frequently co-occurred on plasmids that lacked known backbone replicon annotations, such as *aph(3″)-Ib* and *aph(6)-Id*, CmlA9 and *aadA6*, or *aac(6′)-Ib3* and *aph(3′)-XV*.

Previous studies have reported that strong associations between specific ARGs or antibiotic classes and a specific plasmid taxonomic unit are rarely observed [11,28,34]. This may be due to multi-species analyses, which can mask associations present within individual species. Another important possibility is that plasmid databases are far from comprehensive, with most annotations focusing on clinically relevant or well-studied plasmid types. Consequently, unannotated plasmids or plasmid fragments may have been underexplored. This second explanation is supported by our finding that plasmids annotated with a replicon in this study represent only a small fraction of the predicted plasmid sequences (Figure 4A). Furthermore, although our dataset includes draft genomes and some plasmids may be fragmented, the use of MOB-suite together with reference-based comparison likely reduced false-positive plasmid assignments, making our interpretation relatively conservative. Based on this, future studies could focus on plasmids that have not yet been fully sequenced or assembled, which may provide further insights into plasmid-specific ARGs across additional species.

When constructing the bipartite network, plasmids from *P. aeruginosa* genomes were mapped to reference plasmids in the database based on the smallest Mash distance. Interestingly, many plasmids had closest matches in MOB-suite to reference plasmids originally isolated from or annotated with *Escherichia coli* as the host, and some matched reference plasmids annotated with *Klebsiella pneumoniae* as the host in NCBI (Figure S2). One explanation for this pattern is the presence of broad-host-range plasmids [35], such as IncP, IncQ, and IncN types. Another possibility is that transposon- or integron-containing plasmids are prevalent in hospital, wastewater, or environmental samples where *P. aeruginosa*, *K. pneumoniae*, and *E. coli* may coexist, potentially facilitating plasmid exchange [36,37]. Alternatively, these host annotations may reflect the overrepresentation of *K. pneumoniae* and *E. coli* plasmids in reference databases rather than true host specificity [11,28,34].

Another interesting observation was the identification of *mexD* associated with MGEs, including plasmids, ICEs, IMEs, and prophage regions (Figure 2 and Figure 6B). In *P. aeruginosa*, *mexD* is a chromosomally encoded component of the MexCD-OprJ RND efflux system [9,38]. It is generally considered part of the intrinsic resistance repertoire rather than a horizontally acquired ARG [38]. The presence of *mexD*-related sequences on MGEs raises two non-mutually exclusive possibilities: (i) mobilization of efflux pump-associated genes or gene fragments and (ii) annotation ambiguity due to the high conservation of RND transporter family members across bacterial species. Manual inspection of the raw annotations revealed that RGI classified sequences as *mexD*, whereas ResFinder classified them as *tmexD2*-like genes with approximately 81% sequence identity and 98% coverage. The *tmexCD*-*toprJ*-type efflux systems have recently been described as plasmid-borne genes that contribute to tigecycline resistance in *Enterobacterales* and are phylogenetically related to chromosomal MexCD-OprJ efflux pumps [39]. However, due to the absence of experimental validation and the potential for conserved domain-driven annotation discrepancies, it is unclear whether these sequences represent true *tmexD2*-like variants in *P. aeruginosa* or divergent chromosomal *mexD* homologs located within predicted MGE regions [40,41,42,43]. Further phylogenetic and synteny-based analyses are required to determine if these MGE-associated sequences reflect the horizontal transfer of RND efflux components or are annotation artifacts caused by the evolutionary conservation of efflux pump gene families.

Finally, we detected an abundance of *sul1* on the chromosome, ICEs, and plasmids (Figure 1, Figure 3 and Figure 6A). This finding is consistent with the widespread distribution of *sul1* in *P. aeruginosa* genomes [11,27,28,44]. ICEs are conjugative and chromosomally integrated [29], and because the systematic genomic profiling of ICEs has only recently become widespread, some previously reported chromosomal *sul1* genes may have been located within integrated ICEs or ICE-like regions. The *sul1* gene is best known as part of class 1 integrons, which are DNA elements capable of capturing and expressing gene cassettes, but class 1 integrons are not self-mobilizing and their mobility depends on association with mobile elements such as transposons, plasmids, or ICEs [27,44,45]. Many class 1 integrons reside on plasmids, leveraging plasmid replication and transfer abilities [44,45], while others integrate into ICEs on the chromosome, enabling conjugative transfer [29,46]. In our dataset, the presence of *sul1*, as an integron-associated marker, suggests that integron-associated regions may occur on plasmids and ICEs in *P. aeruginosa*. These class 1 integrons often carry multiple ARGs [44,45], explaining the co-occurrence patterns observed in our analysis. Moreover, insertion sequences (IS elements) frequently co-occur with integrons [27,47]; our study did not assess integrons or IS elements using dedicated detection tools, representing a limitation that future research could address.

We also note that our observations of ARG distribution across different MGEs reflect associations rather than direct evidence that MGEs preferentially acquire specific ARGs. Other limitations of this study include the large fraction of plasmids that could not be annotated to a known backbone replicon, which is currently an unavoidable challenge in plasmid research. We expect that more comprehensive database annotations will facilitate systematic plasmid studies in specific species. Additionally, because long-read sequencing has only become widely adopted over the past decade, many previously submitted genomes are incomplete, presenting challenges for plasmid sequence completeness. As more long-read assemblies yield complete bacterial genomes, future large-scale analyses will benefit from higher-quality datasets.

## 4. Method

We parsed the Genome Taxonomy Database (GTDB; https://gtdb.ecogenomic.org, accessed on 8 April 2025) [48] release 226 to identify genomes belonging to *P. aeruginosa*. GTDB applies independent quality control using CheckM [49], retaining only assemblies with completeness ≥ 50%, contamination < 5%, and a quality score (completeness—5 × contamination) ≥ 50. To maximize genomic coverage, we did not apply additional filtering. All currently available genomes within the GTDB species cluster represented by RS_GCF_001457615.1 were downloaded from the NCBI GenBank and RefSeq FTP site (https://ftp.ncbi.nlm.nih.gov, accessed on 13 April 2026), excluding any assemblies that had been deprecated or removed. These genome assemblies originated from diverse sources, including clinical, environmental, and animal isolates, according to the NCBI metadata provided in GTDB. Sample isolation types were manually classified based on the NCBI isolation source metadata. Sequence types (STs) were assigned using mlst v2.33.1 with the *paeruginosa* scheme (https://github.com/tseemann/mlst, accessed on 12 June 2026) [50], which is based on the seven housekeeping genes: *acsA*, *aroE*, *guaA*, *mutL*, *nuoD*, *ppsA*, and *trpE*. Bacterial genes were predicted using pyrodigal-gv v0.3.2 integrated in geNomad v1.11.0 with database v1.9 and default parameters [51]. Prophages were identified using geNomad. Plasmids were reconstructed using MOB-suite v3.1.9 (mob_recon) [52]. MOB-suite reconstructs plasmid sequences from genome assemblies by identifying plasmid-associated markers and clustering contigs based on sequence similarity and mobilization-related features. It also classifies plasmids into MOB families using a reference relaxase database, providing both sequence reconstruction and functional annotation. ICEs and IMEs were identified via ICEfinder-opt (https://github.com/guogenglin/icefinder-opt, accessed on 16 April 2026), which is an optimized and enhanced local version of ICEfinder2 [53]. ICEfinder-opt first scans bacterial genomes for integrase genes and conserved attachment sites (*attL*/*attR*), then checks the surrounding regions for conjugation-related genes and accessory modules. Elements that carry all conjugation genes are classified as ICEs, while those with only mobilization genes are classified as IMEs. ICE/IME candidates lacking *att* site or tRNA annotations were excluded. This was done to avoid misidentifying plasmids as ICEs/IMEs, given that they share many genes, whereas many chromosomally integrated ICEs are associated with *att* site sequences and are often located at or near tRNA genes. Phage satellites were identified by SatelliteFinder v0.9.1 [54]. SatelliteFinder uses a combination of homology searches and gene neighborhood analysis to identify satellite-specific genes (e.g., capsid or integrase-like genes) and verifies their association with known helper phages based on co-occurrence and conserved genomic composition.

ARGs were identified using Resistance Gene Identifier (RGI) v6.0.5 with the *include_nudge* option and default parameters, CARD version 4.0.1 [55], as well as ResFinder v4.0 with default parameters [56]. RGI uses a curated reference database of ARG protein families and applies sequence alignment to match query sequences to known resistance genes. ResFinder performs nucleotide-level alignment of input sequences against a curated ARG reference set, identifying both acquired and chromosomal resistance genes. For genes predicted by both RGI and ResFinder, if a gene matched multiple ARG families, we retained the annotation with the highest bitscore (for RGI) or identity score (for ResFinder).

For the co-localization network of plasmid-borne ARGs, we first considered each primary cluster reconstructed by MOB-suite per genome as a single plasmid. ARG families assigned to the same primary cluster within a genome were treated as co-located on the same plasmid. The weight between two ARG families was the number of times they co-occurred on plasmids across all *P. aeruginosa* genomes. For the plasmid–ARG bipartite network, we first mapped the identified plasmids to the nearest neighbor in the database via the Mash distance calculated by MOB-suite. This was because the primary clusters are identified independently and were not comparable between genomes. In this case, the nearest neighbors were used as reference-based proxy annotations. This approach also reduced complexity by grouping highly similar plasmids from different genomes into the same representative node. Finally, we used the number of ARGs that the plasmids contained as the weight of the network for each ARG family. The networks were visualized using Gephi v0.10.1 and Gephi-lite v1.0.2 (https://gephi.org/, accessed on 7 May 2026) with force-directed layout. The force-directed parameters were 0.0005 for attraction, 2.5 for repulsion, 0.0001 for gravity, 0.6 for inertia, and 200 for maximum movement. For the bipartite network, edge thickness was the number of associated plasmid–ARG family pairs. Singletons were not included in the figures.

## Data Availability

The datasets generated and analyzed during the current study are available in the Figshare repository at DOI: 10.6084/m9.figshare.32314083.

## Supplementary Materials

The following supporting information can be downloaded at https://www.mdpi.com/article/10.3390/ijms27135938/s1.

## References

[1] De Oliveira D.M.P., Forde B.M., Kidd T.J., Harris P.N.A., Schembri M.A., Beatson S.A., Paterson D.L., Walker M.J. (2020). Antimicrobial Resistance in ESKAPE Pathogens. Clin. Microbiol. Rev..

[2] Miller W.R., Arias C.A. (2024). ESKAPE pathogens: Antimicrobial resistance, epidemiology, clinical impact and therapeutics. Nat. Rev. Microbiol..

[3] Brinkman F.S.L., Winsor G.L., Done R.E., Filloux A., Francis V.I., Goldberg J.B., Greenberg E.P., Han K., Hancock R.E.W., Haney C.H. (2021). The *Pseudomonas aeruginosa* whole genome sequence: A 20th anniversary celebration. Adv. Microb. Physiol..

[4] Bassetti M., Vena A., Croxatto A., Righi E., Guery B. (2018). How to manage *Pseudomonas aeruginosa* infections. Drugs Context.

[5] Reynolds D., Kollef M. (2021). The Epidemiology and Pathogenesis and Treatment of *Pseudomonas aeruginosa* Infections: An Update. Drugs.

[6] Ho C.S., Wong C.T.H., Aung T.T., Lakshminarayanan R., Mehta J.S., Rauz S., McNally A., Kintses B., Peacock S.J., de la Fuente-Nunez C. (2025). Antimicrobial resistance: A concise update. Lancet Microbe.

[7] Horcajada J.P., Montero M., Oliver A., Sorli L., Luque S., Gomez-Zorrilla S., Benito N., Grau S. (2019). Epidemiology and Treatment of Multidrug-Resistant and Extensively Drug-Resistant *Pseudomonas aeruginosa* Infections. Clin. Microbiol. Rev..

[8] Wu W., Huang J., Xu Z. (2024). Antibiotic influx and efflux in *Pseudomonas aeruginosa*: Regulation and therapeutic implications. Microb. Biotechnol..

[9] Fraud S., Campigotto A.J., Chen Z., Poole K. (2008). MexCD-OprJ multidrug efflux system of *Pseudomonas aeruginosa*: Involvement in chlorhexidine resistance and induction by membrane-damaging agents dependent upon the AlgU stress response sigma factor. Antimicrob. Agents Chemother..

[10] Khedkar S., Smyshlyaev G., Letunic I., Maistrenko O.M., Coelho L.P., Orakov A., Forslund S.K., Hildebrand F., Luetge M., Schmidt T.S.B. (2022). Landscape of mobile genetic elements and their antibiotic resistance cargo in prokaryotic genomes. Nucleic Acids Res..

[11] Partridge S.R., Kwong S.M., Firth N., Jensen S.O. (2018). Mobile Genetic Elements Associated with Antimicrobial Resistance. Clin. Microbiol. Rev..

[12] Benler S., Koonin E.V. (2022). Recruitment of Mobile Genetic Elements for Diverse Cellular Functions in Prokaryotes. Front. Mol. Biosci..

[13] Beamud B., Benz F., Bikard D. (2024). Going viral: The role of mobile genetic elements in bacterial immunity. Cell Host Microbe.

[14] Garcillan-Barcia M.P., de la Cruz F., Rocha E.P.C. (2025). The extended mobility of plasmids. Nucleic Acids Res..

[15] Sastre-Dominguez J., Rodera-Fernandez P., DelaFuente J., Martinez-Gonzalez S., Quesada S., Valencoso-Requena M., Calvo-Villamanan A., Costas C., Fuentes-Hernandez A., Santos-Lopez A. (2026). Plasmids promote antimicrobial resistance through insertion sequence-mediated gene inactivation. Nat. Microbiol..

[16] Toribio-Celestino L., Calvo-Villamanan A., Herencias C., Alonso-Del Valle A., Sastre-Dominguez J., Quesada S., Mazel D., Rocha E.P.C., Fernandez-Calvet A., San Millan A. (2024). A plasmid-chromosome crosstalk in multidrug resistant enterobacteria. Nat. Commun..

[17] Hernandez-Beltran J.C.R., Rodriguez-Beltran J., Aguilar-Luviano O.B., Velez-Santiago J., Mondragon-Palomino O., MacLean R.C., Fuentes-Hernandez A., San Millan A., Pena-Miller R. (2024). Plasmid-mediated phenotypic noise leads to transient antibiotic resistance in bacteria. Nat. Commun..

[18] DelaFuente J., Toribio-Celestino L., Santos-Lopez A., Leon-Sampedro R., Alonso-Del Valle A., Costas C., Hernandez-Garcia M., Cui L., Rodriguez-Beltran J., Bikard D. (2022). Within-patient evolution of plasmid-mediated antimicrobial resistance. Nat. Ecol. Evol..

[19] DelaFuente J., Diaz-Colunga J., Sanchez A., San Millan A. (2024). Global epistasis in plasmid-mediated antimicrobial resistance. Mol. Syst. Biol..

[20] Botelho J., Cazares A., Schulenburg H. (2023). The ESKAPE mobilome contributes to the spread of antimicrobial resistance and CRISPR-mediated conflict between mobile genetic elements. Nucleic Acids Res..

[21] Martinez J.L., Coque T.M., Baquero F. (2015). What is a resistance gene? Ranking risk in resistomes. Nat. Rev. Microbiol..

[22] Freschi L., Vincent A.T., Jeukens J., Emond-Rheault J.G., Kukavica-Ibrulj I., Dupont M.J., Charette S.J., Boyle B., Levesque R.C. (2019). The *Pseudomonas aeruginosa* Pan-Genome Provides New Insights on Its Population Structure, Horizontal Gene Transfer, and Pathogenicity. Genome Biol. Evol..

[23] Botelho J., Grosso F., Peixe L. (2019). Antibiotic resistance in *Pseudomonas aeruginosa*—Mechanisms, epidemiology and evolution. Drug Resist. Updates.

[24] Domingues S., da Silva G.J., Nielsen K.M. (2012). Integrons: Vehicles and pathways for horizontal dissemination in bacteria. Mob. Genet. Elem..

[25] Queenan A.M., Bush K. (2007). Carbapenemases: The versatile beta-lactamases. Clin. Microbiol. Rev..

[26] Davies J., Davies D. (2010). Origins and evolution of antibiotic resistance. Microbiol. Mol. Biol. Rev..

[27] Cambray G., Guerout A.M., Mazel D. (2010). Integrons. Annu. Rev. Genet..

[28] Rozwandowicz M., Brouwer M.S.M., Fischer J., Wagenaar J.A., Gonzalez-Zorn B., Guerra B., Mevius D.J., Hordijk J. (2018). Plasmids carrying antimicrobial resistance genes in Enterobacteriaceae. J. Antimicrob. Chemother..

[29] Wozniak R.A., Waldor M.K. (2010). Integrative and conjugative elements: Mosaic mobile genetic elements enabling dynamic lateral gene flow. Nat. Rev. Microbiol..

[30] Cury J., Touchon M., Rocha E.P.C. (2017). Integrative and conjugative elements and their hosts: Composition, distribution and organization. Nucleic Acids Res..

[31] Guglielmini J., Quintais L., Garcillan-Barcia M.P., de la Cruz F., Rocha E.P. (2011). The repertoire of ICE in prokaryotes underscores the unity, diversity, and ubiquity of conjugation. PLoS Genet..

[32] Colomer-Lluch M., Jofre J., Muniesa M. (2011). Antibiotic resistance genes in the bacteriophage DNA fraction of environmental samples. PLoS ONE.

[33] Chan B.K., Sistrom M., Wertz J.E., Kortright K.E., Narayan D., Turner P.E. (2016). Phage selection restores antibiotic sensitivity in MDR *Pseudomonas aeruginosa*. Sci. Rep..

[34] Orlek A., Stoesser N., Anjum M.F., Doumith M., Ellington M.J., Peto T., Crook D., Woodford N., Walker A.S., Phan H. (2017). Plasmid Classification in an Era of Whole-Genome Sequencing: Application in Studies of Antibiotic Resistance Epidemiology. Front. Microbiol..

[35] Smillie C., Garcillan-Barcia M.P., Francia M.V., Rocha E.P., de la Cruz F. (2010). Mobility of plasmids. Microbiol. Mol. Biol. Rev..

[36] Smillie C.S., Smith M.B., Friedman J., Cordero O.X., David L.A., Alm E.J. (2011). Ecology drives a global network of gene exchange connecting the human microbiome. Nature.

[37] Moura A., Oliveira C., Henriques I., Smalla K., Correia A. (2012). Broad diversity of conjugative plasmids in integron-carrying bacteria from wastewater environments. FEMS Microbiol. Lett..

[38] Gomis-Font M.A., Pitart C., Del Barrio-Tofino E., Zboromyrska Y., Cortes-Lara S., Mulet X., Marco F., Vila J., Lopez-Causape C., Oliver A. (2021). Emergence of Resistance to Novel Cephalosporin-β-Lactamase Inhibitor Combinations through the Modification of the *Pseudomonas aeruginosa* MexCD-OprJ Efflux Pump. Antimicrob. Agents Chemother..

[39] Dong N., Zeng Y., Wang Y., Liu C., Lu J., Cai C., Liu X., Chen Y., Wu Y., Fang Y. (2022). Distribution and spread of the mobilised RND efflux pump gene cluster *tmexCD-toprJ* in clinical Gram-negative bacteria: A molecular epidemiological study. Lancet Microbe.

[40] Wang C.Z., Gao X., Liang X.H., Lv L.C., Lu L.T., Yue C., Cui X.X., Yang K.E., Lu D., Liu J.H. (2023). Pseudomonas Acts as a Reservoir of Novel Tigecycline Resistance Efflux Pump *tmexC6D6-toprJ1b* and *tmexCD-toprJ* Variants. Microbiol. Spectr..

[41] Wang C., Gao X., Zhang X., Yue C., Lv L., Lu L., Liu J.H. (2025). Emergence of two novel *tmexCD-toprJ* subtypes mediating tigecycline resistance in the megaplasmids from *Pseudomonas putida*. Microbiol. Res..

[42] Wu Y., Zhuang Y., Wu C., Jia H., He F., Ruan Z. (2024). Global emergence of Gram-negative bacteria carrying the mobilised RND-type efflux pump gene cluster *tmexCD-toprJ* variants. Lancet Microbe.

[43] Wan L., Li X., Zheng X., Chen T., Yang Y., Chen Y., Liu X., Wang C. (2026). Genomic insights into the *tmexCD-toprJ*: Plasmid-mediated evolution, dissemination and diversity in bacterial populations. J. Antimicrob. Chemother..

[44] Fluit A.C., Schmitz F.J. (1999). Class 1 integrons, gene cassettes, mobility, and epidemiology. Eur. J. Clin. Microbiol. Infect. Dis..

[45] Partridge S.R., Tsafnat G., Coiera E., Iredell J.R. (2009). Gene cassettes and cassette arrays in mobile resistance integrons. FEMS Microbiol. Rev..

[46] Johnson C.M., Grossman A.D. (2015). Integrative and Conjugative Elements (ICEs): What They Do and How They Work. Annu. Rev. Genet..

[47] Mazel D. (2006). Integrons: Agents of bacterial evolution. Nat. Rev. Microbiol..

[48] Parks D.H., Chuvochina M., Chaumeil P.A., Rinke C., Mussig A.J., Hugenholtz P. (2020). A complete domain-to-species taxonomy for Bacteria and Archaea. Nat. Biotechnol..

[49] Parks D.H., Imelfort M., Skennerton C.T., Hugenholtz P., Tyson G.W. (2015). CheckM: Assessing the quality of microbial genomes recovered from isolates, single cells, and metagenomes. Genome Res..

[50] Jolley K.A., Bray J.E., Maiden M.C.J. (2018). Open-access bacterial population genomics: BIGSdb software, the PubMLST.org website and their applications. Wellcome Open Res..

[51] Camargo A.P., Roux S., Schulz F., Babinski M., Xu Y., Hu B., Chain P.S.G., Nayfach S., Kyrpides N.C. (2024). Identification of mobile genetic elements with geNomad. Nat. Biotechnol..

[52] Robertson J., Nash J.H.E. (2018). MOB-suite: Software tools for clustering, reconstruction and typing of plasmids from draft assemblies. Microb. Genom..

[53] Wang M., Liu G., Liu M., Tai C., Deng Z., Song J., Ou H.Y. (2024). ICEberg 3.0: Functional categorization and analysis of the integrative and conjugative elements in bacteria. Nucleic Acids Res..

[54] de Sousa J.A.M., Fillol-Salom A., Penades J.R., Rocha E.P.C. (2023). Identification and characterization of thousands of bacteriophage satellites across bacteria. Nucleic Acids Res..

[55] Alcock B.P., Huynh W., Chalil R., Smith K.W., Raphenya A.R., Wlodarski M.A., Edalatmand A., Petkau A., Syed S.A., Tsang K.K. (2023). CARD 2023: Expanded curation, support for machine learning, and resistome prediction at the Comprehensive Antibiotic Resistance Database. Nucleic Acids Res..

[56] Bortolaia V., Kaas R.S., Ruppe E., Roberts M.C., Schwarz S., Cattoir V., Philippon A., Allesoe R.L., Rebelo A.R., Florensa A.F. (2020). ResFinder 4.0 for predictions of phenotypes from genotypes. J. Antimicrob. Chemother..

