# Supplementary material for: In Silico Genomic Analysis of Antibiotic Resistance Genes Carried by Mobile Genetic Elements in Pseudomonas aeruginosa
Source: Int J Mol Sci. 2026 Jul 1;27(13):5938. doi: 10.3390/ijms27135938 (PMC13362518; doi:10.3390/ijms27135938)
Supplement: Supplementary file 1 [file ijms-27-05938-s001.zip › Supplementary Files v2/Supplementary Figure S2.pdf]

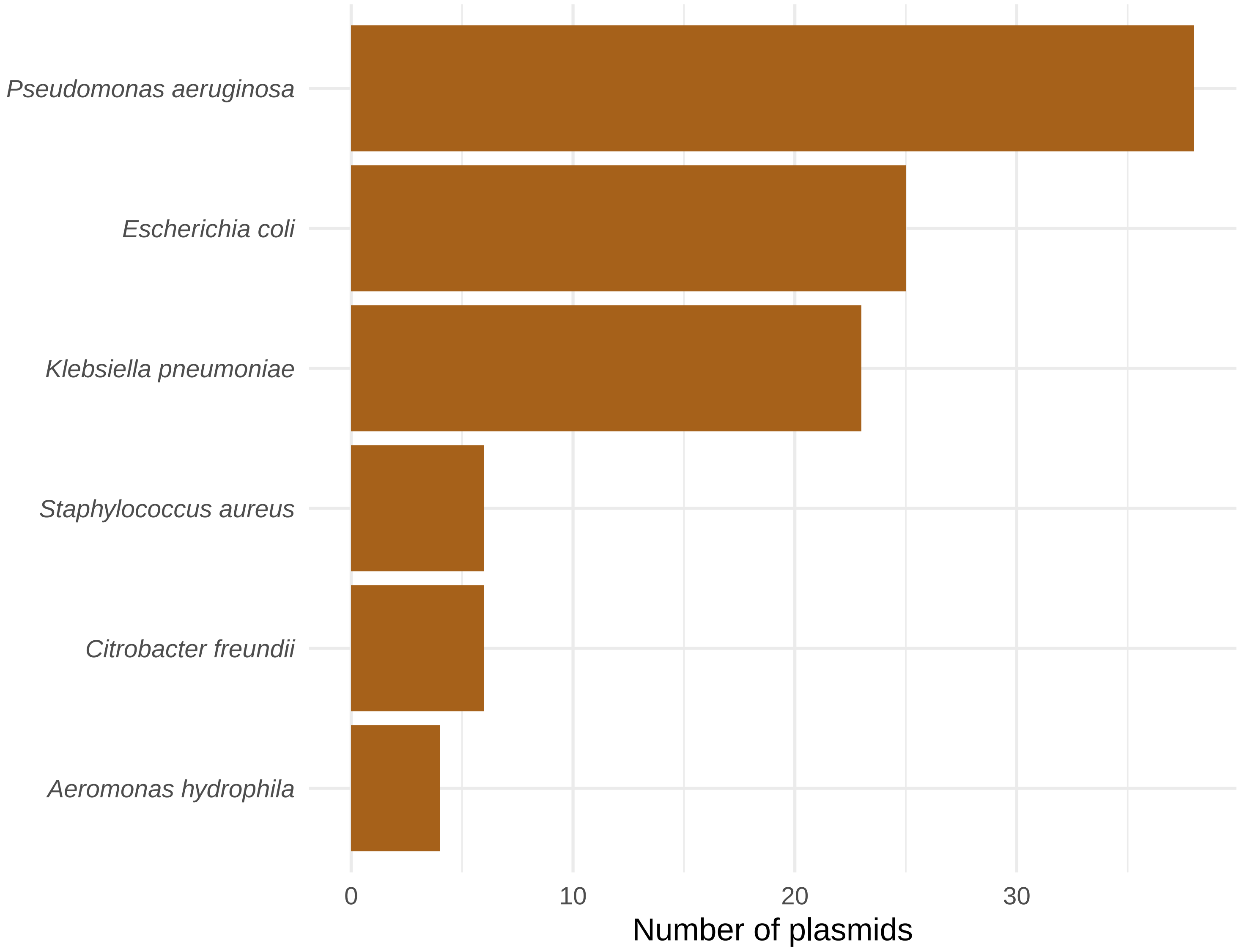
Pseudomonas aeruginosa
Escherichia coli
Klebsiella pneumoniae
Staphylococcus aureus
Citrobacter freundii
Aeromonas hydrophila
0
10
20
30
Number of plasmids
