# Supplementary figures and images for: In Silico Genomic Analysis of Antibiotic Resistance Genes Carried by Mobile Genetic Elements in Pseudomonas aeruginosa
Source: Int J Mol Sci. 2026 Jul 1;27(13):5938. doi: 10.3390/ijms27135938 (PMC13362518; doi:10.3390/ijms27135938)

**A**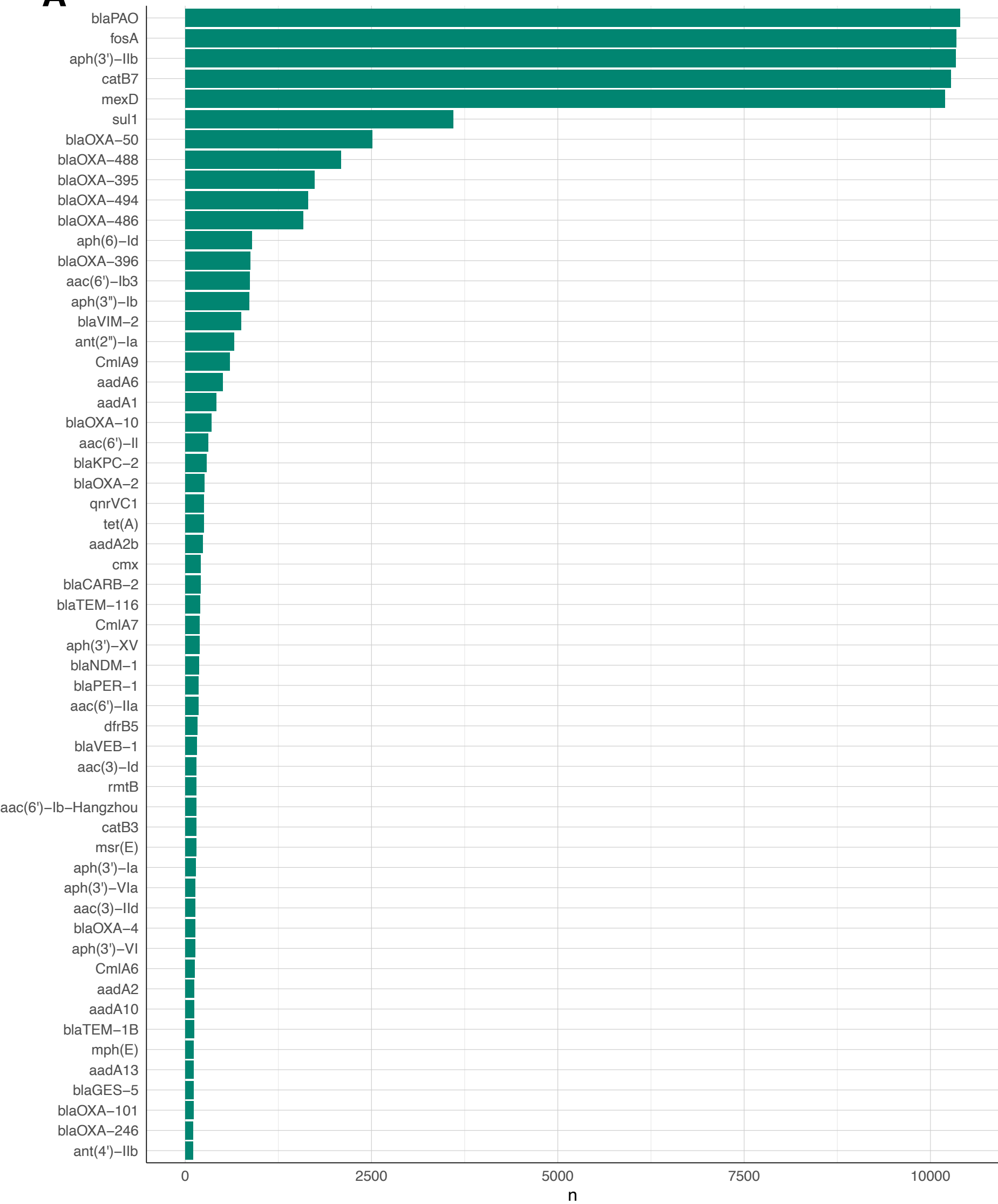**B**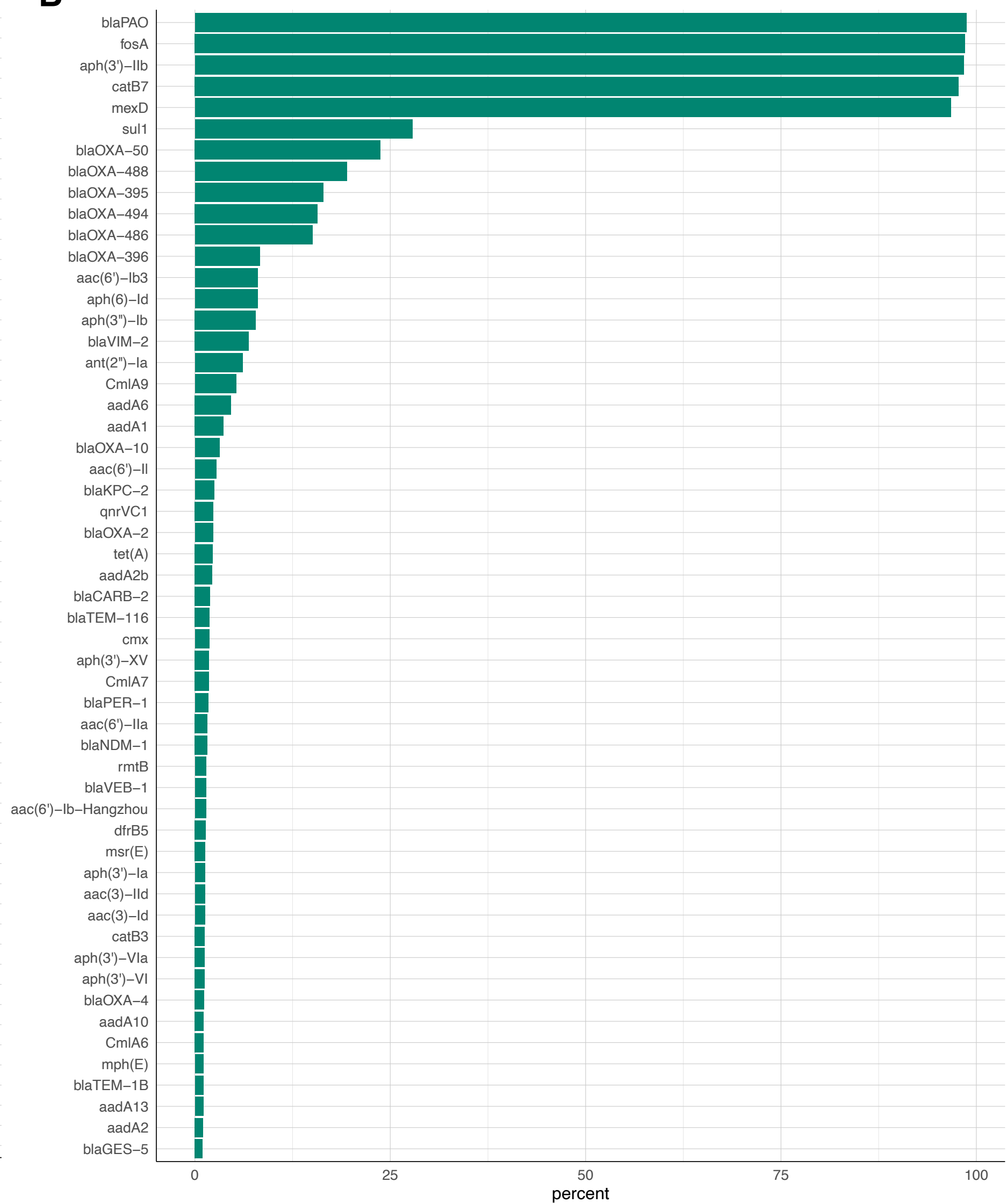

Supplement: Supplementary file 1 [file ijms-27-05938-s001.zip › Supplementary Files v2/Supplementary Figure S1.pdf]
